# Supplementary material for: Global stable-isotope tracing metabolomics reveals system-wide metabolic alternations in aging Drosophila
Source: Nat Commun. 2022 Jun 20;13:3518. doi: 10.1038/s41467-022-31268-6 (PMC9209425; doi:10.1038/s41467-022-31268-6)
Supplement: Supplementary file 1 — Supplementary Information [file 41467_2022_31268_MOESM1_ESM.pdf]

Supplementary Information:

Global Stable-isotope Tracing Metabolomics Reveals System-wide Metabolic Alternations in Aging *Drosophila*

Z. Zhu et al.

List of Supplementary Figures

Supplementary Figure 1: Detailed data processing workflow of MetTracer.

Supplementary Figure 2: Performance evaluation of MetTracer using 293T cell dataset acquired on TripleTOF 6600.

Supplementary Figure 3: Examples to demonstrate the advantages of targeted extraction in MetTracer.

Supplementary Figure 4: Examples to demonstrate the performance of MetTracer for highly labeled metabolites.

Supplementary Figure 5: Example to demonstrate the utilization of PPC score for correct peak grouping in MetTracer.

Supplementary Figure 6: Example to demonstrate the isotope contamination correction in MetTracer.

Supplementary Figure 7: Performance evaluation of MetTracer using 293T cell dataset acquired on Orbitrap Exploris 480.

Supplementary Figure 8: Performance evaluation of MetTracer using the *Drosophila* head tissue.

Supplementary Figure 9: Examples of metabolites in each labeling cluster from head tissue of *Drosophila*.

Supplementary Figure 10: *In vivo* stable-isotope tracing in muscle tissue of *Drosophila*.

Supplementary Figure 11: Comparison of metabolic profiles in different tissues.

Supplementary Figure 12: Metabolic homeostasis is lost during aging in head tissue of *Drosophila*.

Supplementary Figure 13: Alternations of inter-cluster metabolic correlations during aging.

Supplementary Figure 14: Abundance correlation of metabolites and their labeling extents, fractions and rates.

Supplementary Figure 15: Examples of metabolites with different labeling rates in head tissue of *Drosophila*.

Supplementary Figure 16: Comparison of metabolic heterogeneity between 3d and 30d *Drosophila*.

List of Supplementary Tables

Supplementary Table 1: Number of labeled metabolites using four software tools.

Supplementary Table 2: False positive rate evaluation using 293T cell samples acquired on TripleTOF 6600

Supplementary Table 3: False positive rate evaluation using 293T cell samples acquired on Orbitrap Exploris 480.

Supplementary Table 4: False positive rate evaluation of MetTracer using fly head samples acquired on TripleTOF 6600.

Supplementary Table 5: Names of KEGG pathways for *Drosophila.*

Supplementary Table 6: Metabolites correlated with pyruvate in 3d *Drosophila.*

Supplementary Table 7: Comparison of metabolic observations between our study and other studies.

Supplementary Table 8: The parameters for MetTracer.

Supplementary Table 9: The parameters for X^{13}CMS, geoRge and El-MAVEN.

Supplementary Table 10: The modifications of default parameters in different software tools.

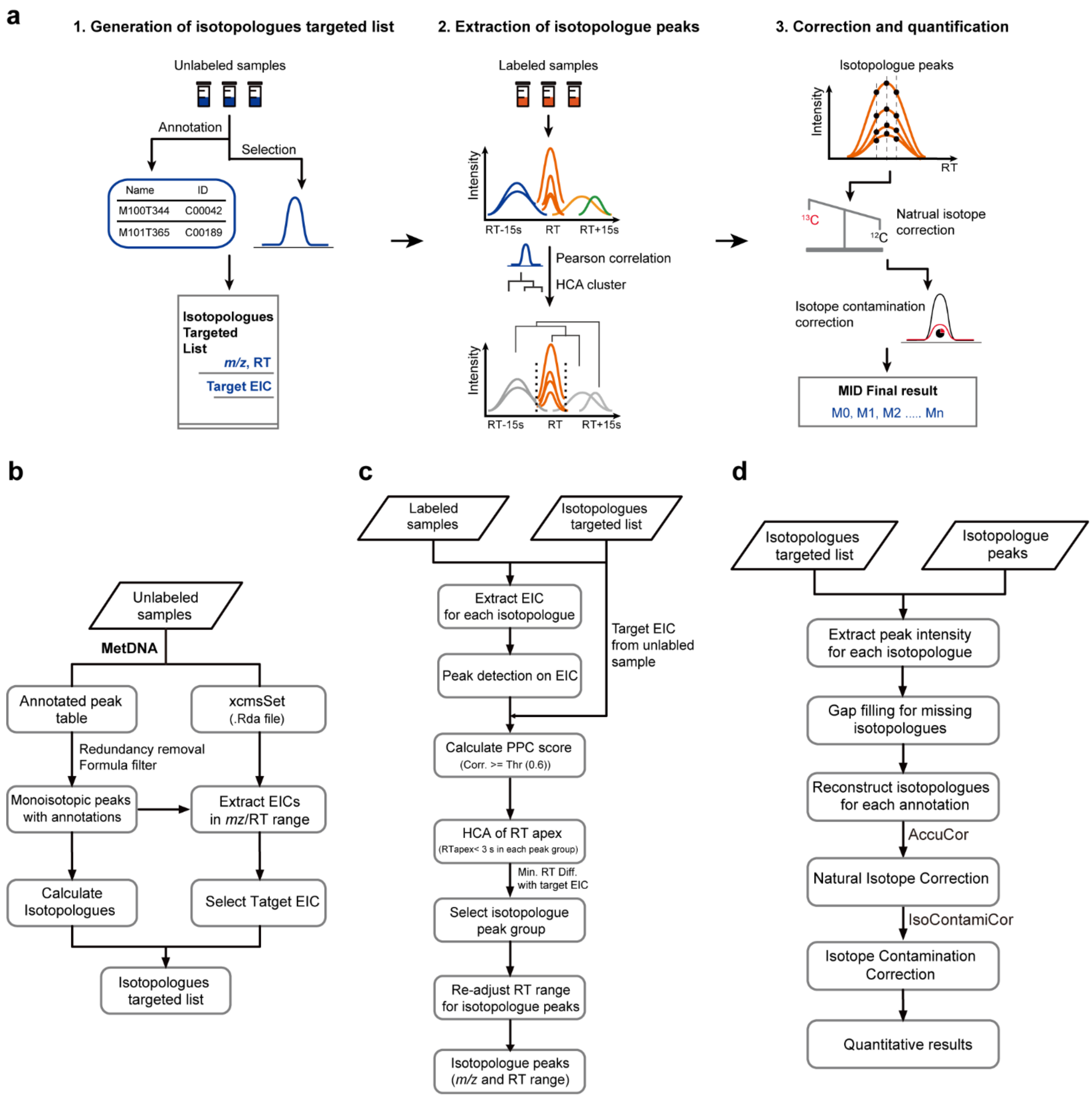

Supplementary Figure 1. Detailed data processing workflow of MetTracer. Related to Figure 1.

(**a**) Schematic illustration of MetTracer. (**b-d**) The detailed workflow of each processing step in panel a.

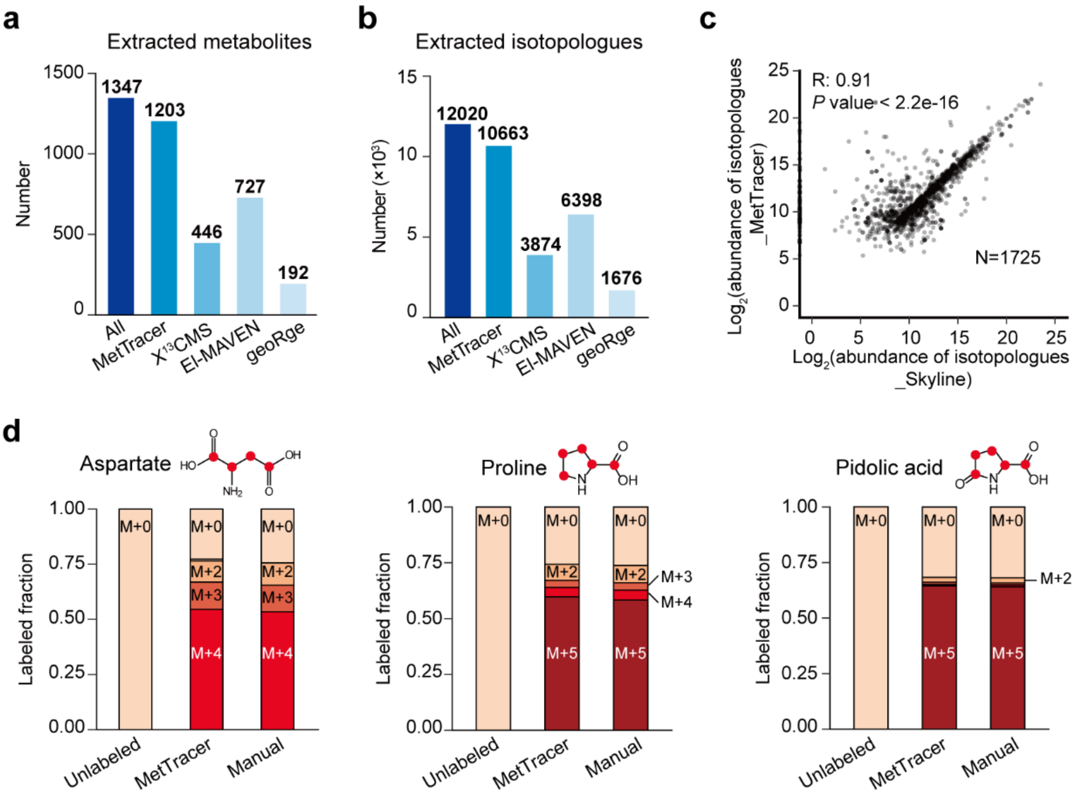

Supplementary Figure 2. Performance evaluation of MetTracer using 293T cell dataset acquired on TripleTOF 6600. Related to Figure 1.

(**a**) Number of extracted metabolites using MetTracer and other software tools. (**b**) Number of extracted isotopologues using MetTracer and other software tools.(**c**) Correlation plot between abundances of labeled isotopologues obtained from MetTracer and Skyline (N = 1725). The *P* value of Pearson correlation coefficient was calculated by two-sided Student's *t*-test. (**d**) Labeled fractions of example metabolites analyzed by MetTracer and Skyline (manual analysis). Source data are provided as a Source Data file.

a

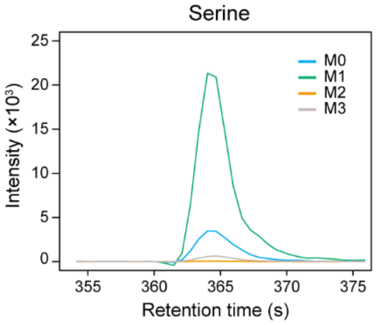

Result in MetTracer

base peak	id	mz	label
M104T365	C00065	104.0342	0
M104T365	C00065	105.0376	1
M104T365	C00065	106.0409	2
M104T365	C00065	107.0443	3

Result in X13CMS

compound	isotopologue	groupID	rt
101.0247	101.0247	452	364.1
	102.0276	470	364.4
	103.0308	484	363.9
	104.0349	495	364.3
	105.0378	499	363.9
	107.0445	522	363.2

b

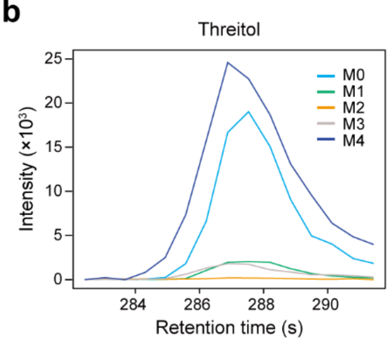

Result in MetTracer

base peak	id	mz	label
M181T288_2	C16884	181.0707	0
M181T288_2	C16884	182.0740	1
M181T288_2	C16884	183.0774	2
M181T288_2	C16884	184.0807	3
M181T288_2	C16884	185.0841	4

Result in geoRge

inc_id	feature_id	mzmed	rtmed	atoms
190	2391	180.0670	287.4	0
190	2422	181.0701	287.4	1
190	2445	182.0725	287.4	2
190	2553	186.0875	287.9	6

Supplementary Figure 3. Examples to demonstrate the advantages of targeted extraction in MetTracer. (a) Left panel, extracted ion chromatograms of isotopologue peaks of serine; middle panel, output results by MetTracer; right panel, output results of the isotopologue peak group containing M104T365 by $X^{13}CMS$. **(b)** Left panel, extracted ion chromatograms of isotopologue peaks of threitol; middle panel, output results by MetTracer; right panel, output results of the isotopologue peak group containing M181T288_2 by geoRge. The examples were from labeled 293T cell samples acquired with the TripleTOF 6600 instrument.

a

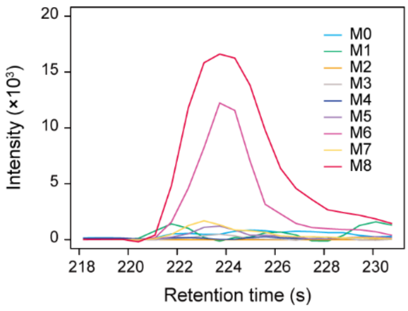

Result in MetTracer

base peak	id	mz	label	sample 1
M221T224	C01061	221.0057	0	0.03
M221T224	C01061	222.0090	1	0.00
M221T224	C01061	223.0124	2	0.00
M221T224	C01061	224.0157	3	0.00
M221T224	C01061	225.0191	4	0.00
M221T224	C01061	226.0224	5	0.01
M221T224	C01061	227.0258	6	0.38
M221T224	C01061	228.0291	7	0.00
M221T224	C01061	229.0325	8	0.58

b

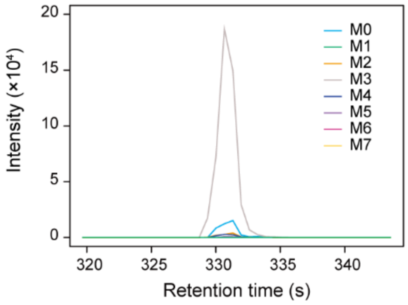

Result in MetTracer

base peak	id	mz	label	sample 1
M170T331	C03871	170.0448	0	0.07
M170T331	C03871	171.0481	1	0.00
M170T331	C03871	172.0515	2	0.00
M170T331	C03871	173.0548	3	0.93
M170T331	C03871	174.0582	4	0.00
M170T331	C03871	175.0616	5	0.00
M170T331	C03871	176.0649	6	0.00
M170T331	C03871	177.0683	7	0.00

Supplementary Figure 4. Examples to demonstrate the performance of MetTracer for highly labeled metabolites. (a) Left panel, extracted ion chromatograms of isotopologue peaks of 4-Fumarylacetoacetate; right panel, output results by MetTracer. **(b)** Left panel, extracted ion chromatograms of isotopologue peaks of 2-Amino-6-oxoheptanedioate; right panel, output results by MetTracer. The examples were from labeled 293T cell samples acquired with the TripleTOF 6600 instrument.

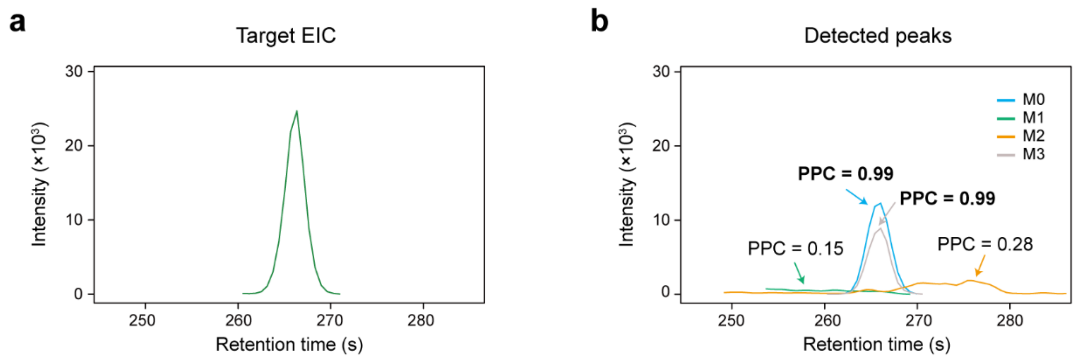

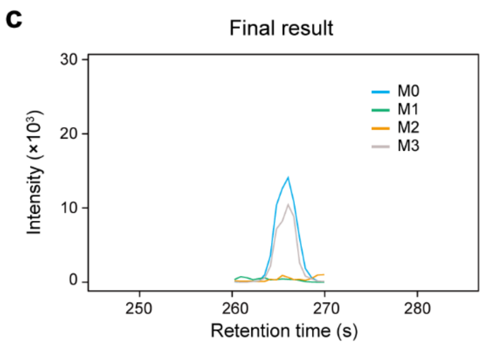

d Result in MetTracer

base peak	id	mz	label	sample 1
M153T266	C00802	152.9907	0	0.57
M153T266	C00802	153.994	1	0.00
M153T266	C00802	154.9974	2	0.01
M153T266	C00802	156.0007	3	0.42

Supplementary Figure 5. Example to demonstrate the utilization of PPC score for correct peak grouping in MetTracer. (a) Targeted EIC of M153T266 recorded in the targeted list. **(b)** Isotopologue peaks extracted by MetTracer and their calculated PPC scores. **(c)** Extracted ion chromatograms of isotopologue peaks of oxalureate finally decided by MetTracer. **(d)** Output results of oxalureate by MetTracer. The examples were from labeled 293T cell samples acquired with the TripleTOF 6600 instrument.

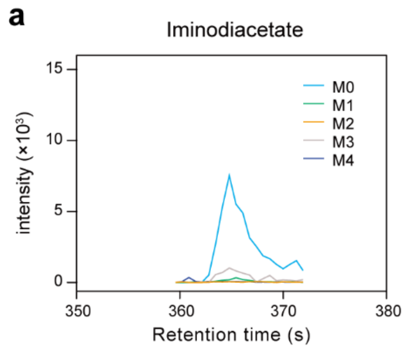

b Result before contamination correction

base peak	id	mz	label	Unlabel sample 1
M114T366	C19911	114.0186	0	0.88
M114T366	C19911	115.0219	1	0.00
M114T366	C19911	116.0253	2	0.00
M114T366	C19911	117.0286	3	0.12
M114T366	C19911	118.0320	4	0.00

c Result after contamination correction

base peak	id	mz	label	Unlabel sample 1
M114T366	C19911	114.0186	0	1.00
M114T366	C19911	115.0219	1	0.00
M114T366	C19911	116.0253	2	0.00
M114T366	C19911	117.0286	3	0.00
M114T366	C19911	118.0320	4	0.00

Supplementary Figure 6. Example to demonstrate the isotope contamination correction in MetTracer. (a) Extracted ion chromatograms of isotopologue peaks of iminodiacetate before contamination correction in unlabeled samples. **(b)** Labeling extent result of iminodiacetate before contamination correction. **(c)** Labeling extent result of iminodiacetate after contamination correction. The examples were from unlabeled 293T cell samples acquired with the TripleTOF 6600 instrument.

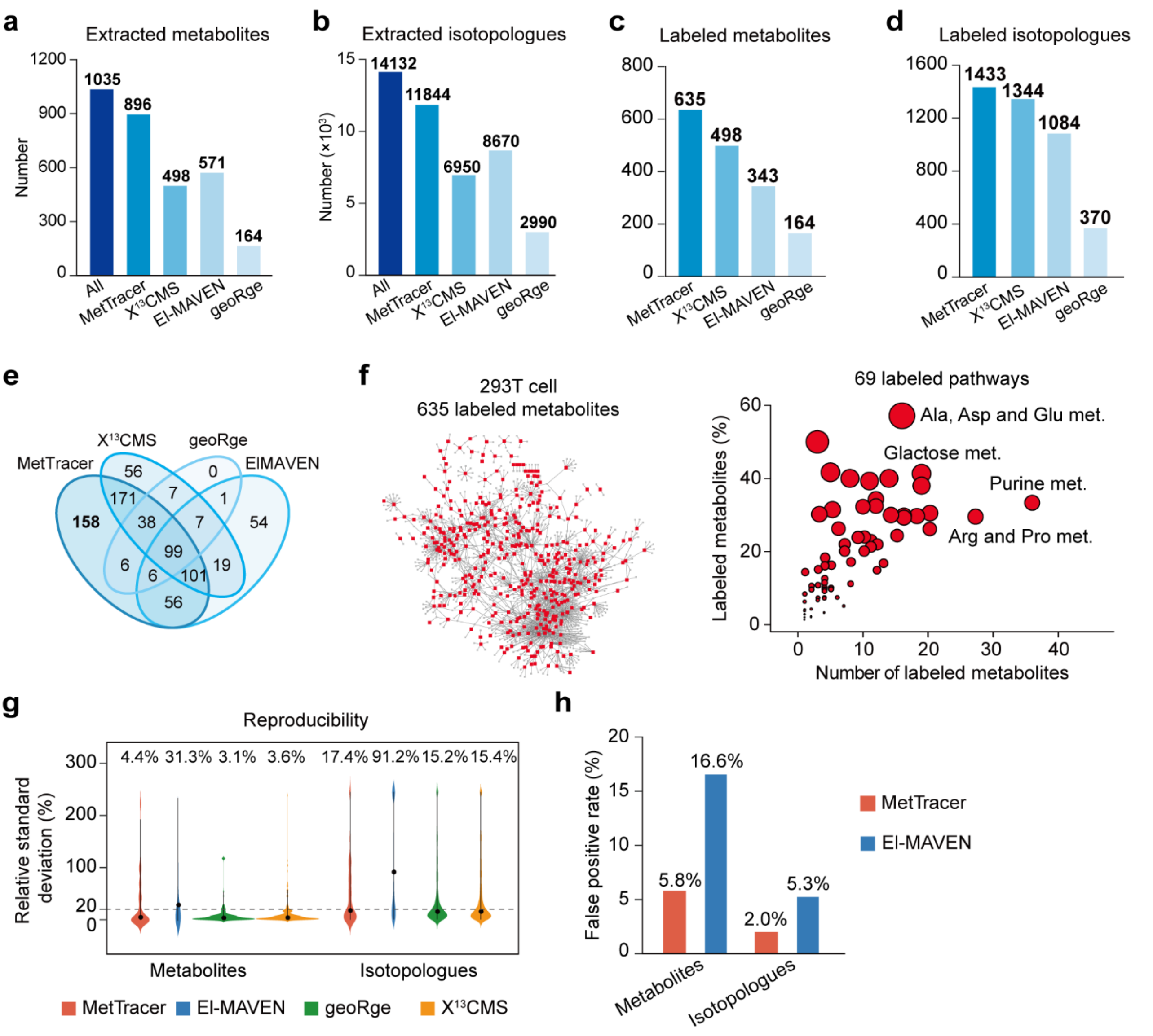

Supplementary Figure 7. Performance evaluation of MetTracer using 293T cell dataset acquired on Orbitrap Exploris 480. Related to Figure 1.

(**a**) Number of extracted metabolites using MetTracer and other indicated software tools. (**b**) Number of extracted isotopologues using MetTracer and other software tools. (**c**) Numbers of labeled metabolites using MetTracer and other software tools. (**d**) Numbers of labeled isotopologues using MetTracer and other software tools. (**e**) The Venn diagram shows the overlap of the labeled metabolites using MetTracer and other software. (**f**) Distributions of the 635 labeled metabolites in 293T cells in the metabolic network (left panel) and pathways (right panel). Red dots in the left panel represent the labeled metabolites. The circle size in the right panel represents the ratio between the labeled metabolites and the entire pathway. (**g**) Relative standard deviation (RSD) distributions of metabolites and isotopologues obtained from MetTracer and other software tools (n=6 technical replicates of 293T cell samples). The black dots represent median RSD. (**h**) False positive rates of the labeled metabolites and isotopologues obtained from MetTracer and El-MAVEN. Source data are provided as a Source Data file.

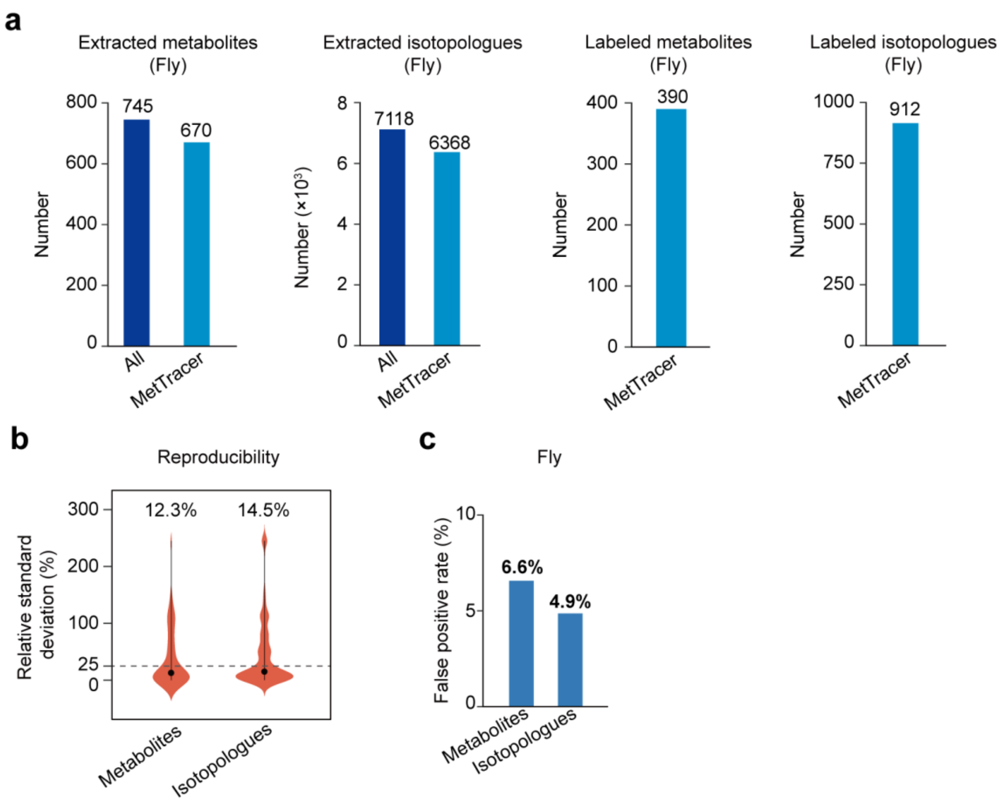

Supplementary Figure 8. Performance evaluation of MetTracer using the *Drosophila* head tissue. Related to Figure 2.

(**a**) Number of extracted metabolites, extracted isotopologues, labeled metabolites and labeled isotopologues in *Drosophila* head tissues using MetTracer and other software tools. (**b**) Relative standard deviation (RSD) distributions of metabolites and isotopologues obtained from MetTracer (n=6 technical replicates of pooled quality control samples for *Drosophila* head tissues). The black dots represent median RSD. (**c**) False positive rates of labeled metabolites and isotopologues obtained from MetTracer. Source data are provided as a Source Data file.

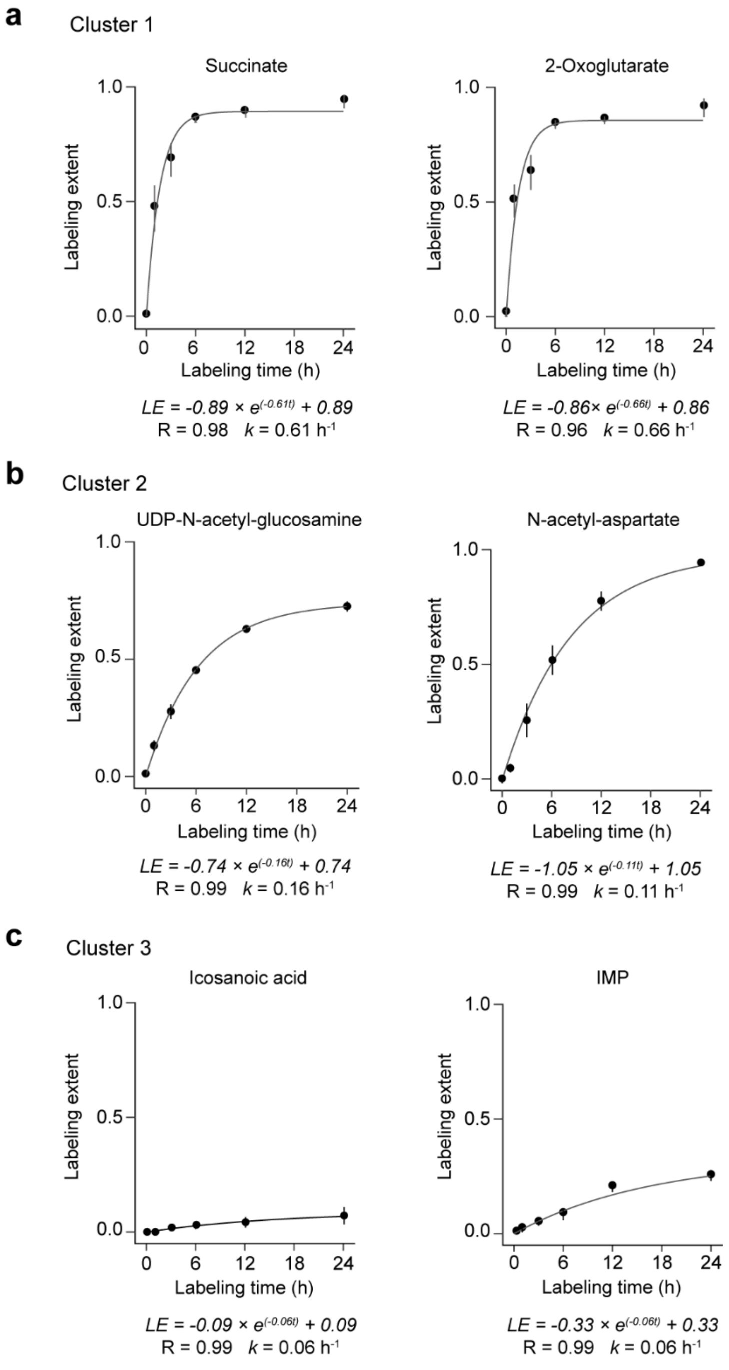

Supplementary Figure 9. Examples of metabolites in each labeling cluster from head tissue of *Drosophila*. Related to Figure 2.

(**a-c**) Examples of metabolites in cluster 1 (**a**), cluster 2 (**b**) and cluster 3 (**c**). Data are presented as median ± SD; n = 10 biological replicates in each time point; grey line, fitting curve. Source data are provided as a Source Data file.

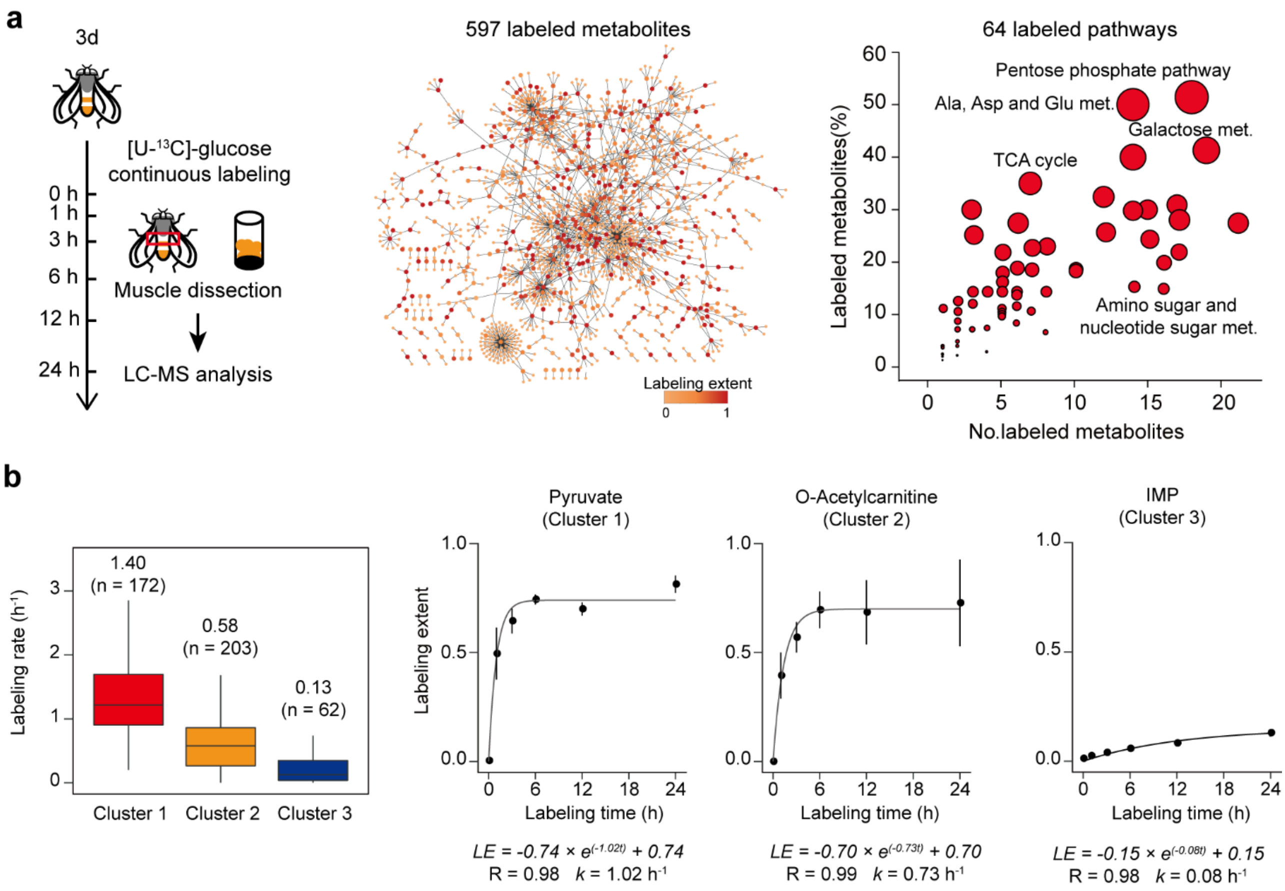

Supplementary Figure 10. *In vivo* stable-isotope tracing in muscle tissue of *Drosophila*. Related to Figure 2.

(**a**) Left panel, schematic illustration of *in vivo* stable-isotope tracing in muscle tissue of *Drosophila*. Middle and right panels, distributions of labeled metabolites (middle panel) and labeled pathways (right panel) in *Drosophila* muscle tissue. (**b**) Left panel, statistics of labeling rates (k values; h^{-1}) of metabolites in 3 labeling clusters in *Drosophila* muscle tissue (n=172, 203, 62 metabolites successfully fitted in each cluster). The centerlines of the box plots indicate the median; the lower and upper lines in box plots correspond to 25th and 75th quartiles, and the whiskers extend to the most extreme data points within 1.5 interquartile ranges (IQR). Right panel, three examples of metabolites from cluster 1, cluster 2 and cluster 3. Data are presented as median ± SD; n = 10 biological replicates in each time point; grey line, fitting curve. Source data are provided as a Source Data file.

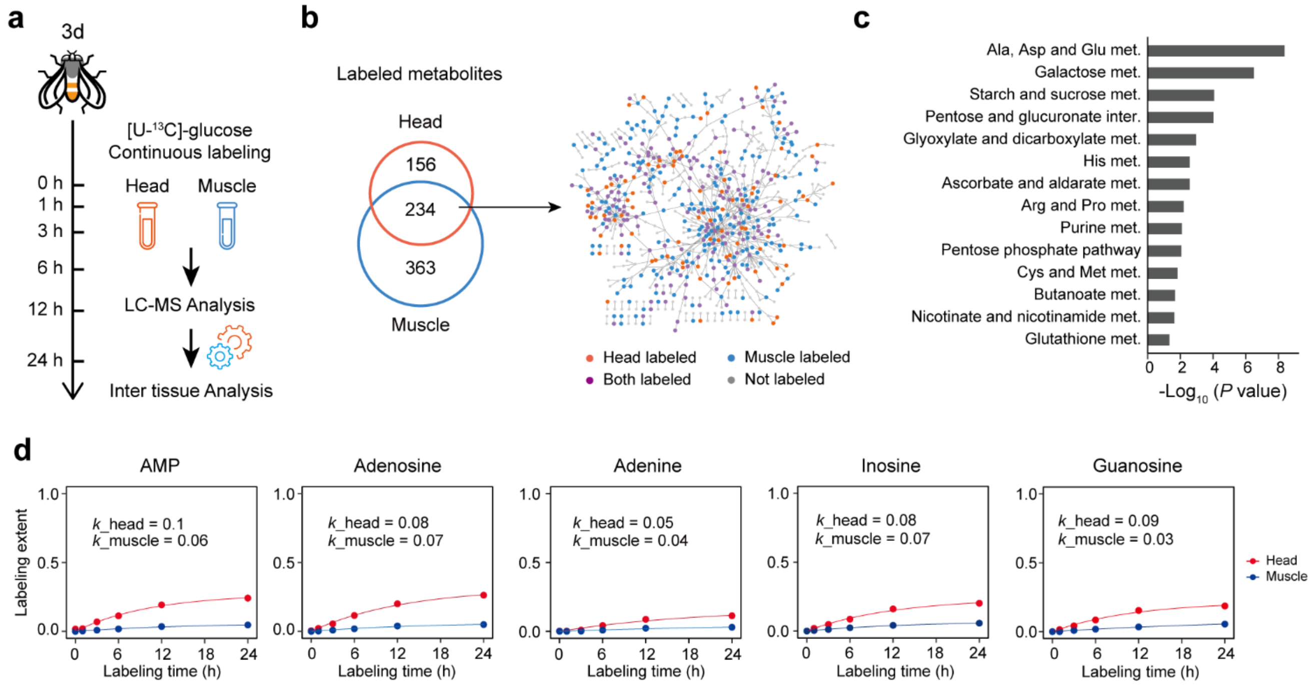

Supplementary Figure 11. Comparison of metabolic profiles in different tissues. Related to Figure 3. (**a**) Schematic illustration of the analysis of multi-tissue metabolic activities. (**b**) Venn diagram showing the number of labeled metabolites in head and muscle tissues. Network diagram showing the labeled metabolites in head and muscle tissues. (**c**) The enriched metabolic pathways for 234 shared metabolites in both of head and muscle tissues. (Hypergeometric test; *P* value < 0.05). For each pathway, a minimum number of labeled metabolites was 5. (**d**) Labeling kinetics of metabolites in purine metabolism. Data are presented as median ± SD; n = 10 per time point per tissue; red and blue lines, fitting curves for head and muscle tissues, respectively. Source data are provided as a Source Data file.

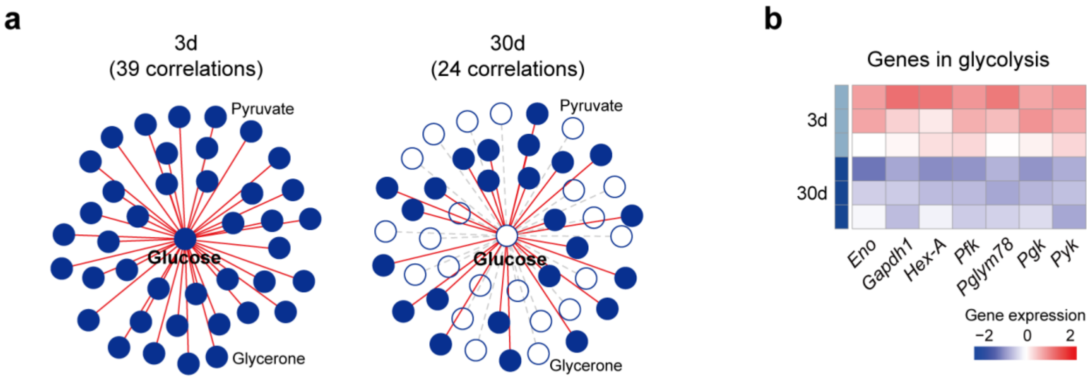

Supplementary Figure 12. Metabolic homeostasis is lost during aging in head tissue of *Drosophila*. Related to Figure 4.

(**a**)The correlation loss between glucose and other metabolites during aging. Red line, significant correlation; grey dashed line, no correlation. (**b**) Decreased mRNA expressions in glycolysis pathway during aging (3d vs. 30d) in head tissue of *Drosophila*.

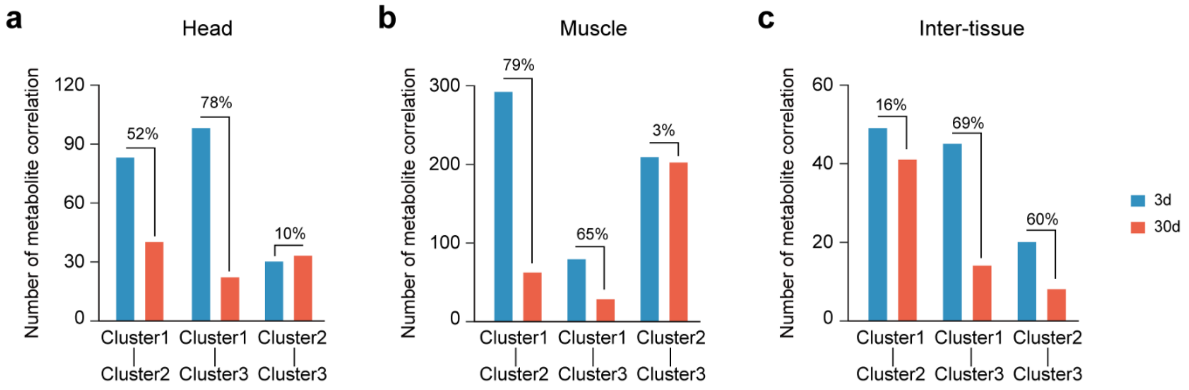

Supplementary Figure 13. Alternations of inter-cluster metabolic correlations during aging. Bar plot showing the numbers of inter-cluster metabolite-metabolite correlations in young and old Drosophila: head tissue **(a)**, muscle tissue **(b)**, inter tissue **(c)**.

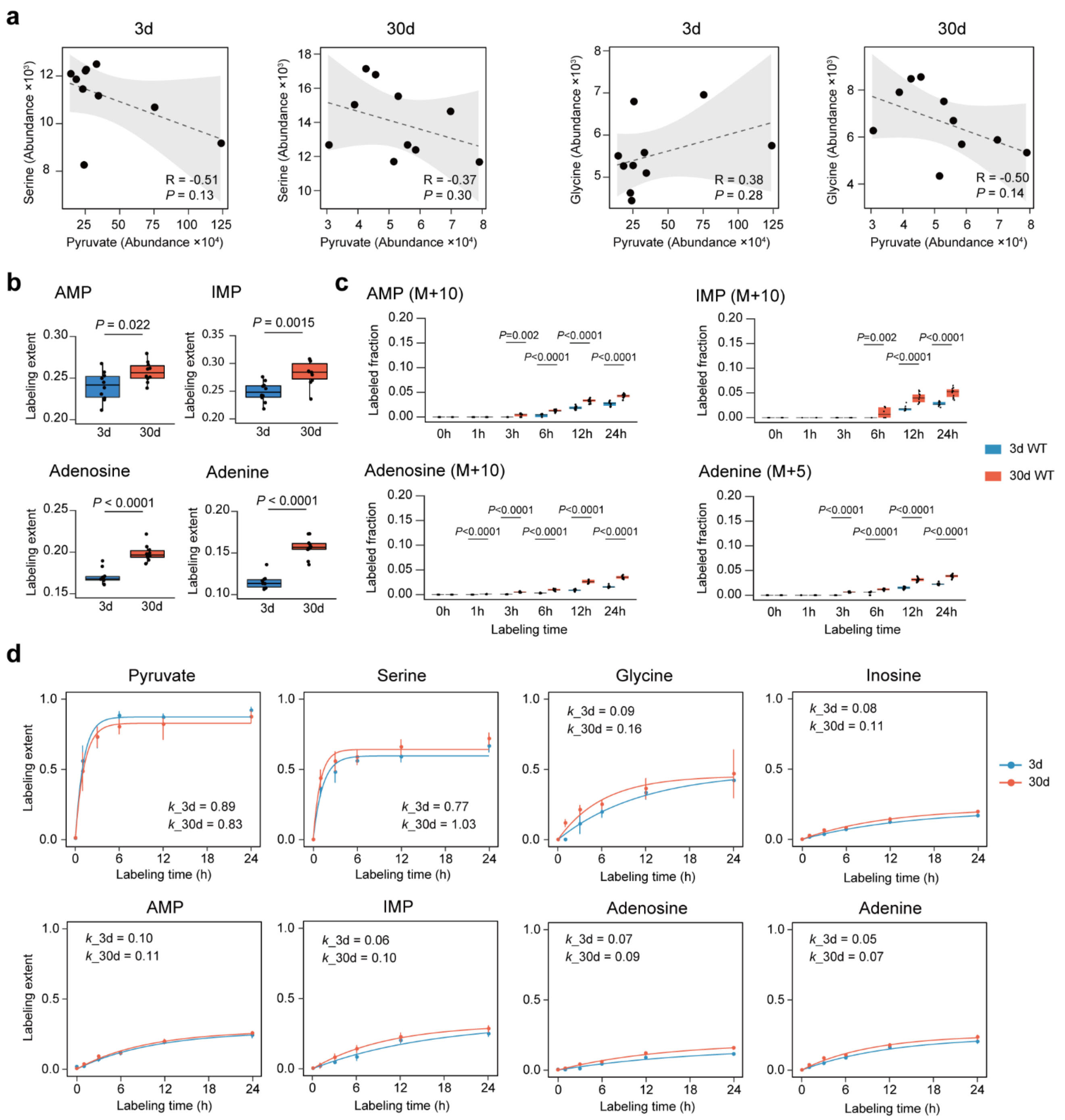

Supplementary Figure 14. Abundance correlation of metabolites and their labeling extents, fractions and rates. Related to Figure 5.

(**a**) Abundance correlations of serine and glycine against pyruvate during aging (n = 10 per group). Dashed line, linear regression; grey shadow, 95% confidence interval of the linear relationship between pyruvate and serine or glycine. The *P* value of Pearson correlation coefficient was calculated by two-sided Student's *t*-test. (**b**) Labeling extents (LE) of metabolites in 3d and 30d *Drosophila* (n = 10 per group; two-tailed Student's *t*-test). (**c**) Boxplots showing the labeling fraction of AMP (M+10), IMP (M+10), adenosine (M+10) and adenine (M+5). The lower, middle, and upper lines in box plots correspond to 25th, 50th, and 75th quartiles, and the whiskers extend to the most extreme data points within 1.5 interquartile ranges (IQR) *P* value < 0.001: ***; *P* value < 0.01: **; *P* value < 0.05: * (n = 10 in each group). Labeled fraction was normalized to the fraction of glucose

(M+6) at the time of head dissection. (**d**) Labeling rates of the related metabolites. Data are presented as median ± SD; n = 10 per time point per tissue; light blue and dark blue lines, fitting curves for 3d and 30d head tissues, respectively. In c, the labeled fraction was normalized to the fraction of glucose (M+6). In b and c, the centerlines of the box plots indicate the median; the lower and upper lines in box plots correspond to 25th and 75th quartiles, and the whiskers extend to the most extreme data points within 1.5 interquartile ranges (IQR). Two-tailed Student's *t*-test. Source data are provided as a Source Data file.

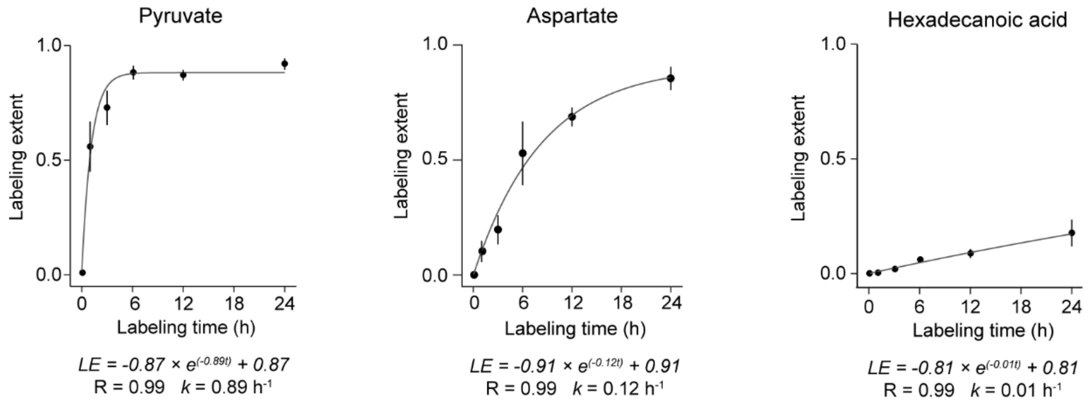

Supplementary Figure 15. Examples of metabolites with different labeling rates in head tissue of *Drosophila*. Data are presented as median ± SD; n = 10 biological replicates in each time point; grey line, fitting curve. Source data are provided as a Source Data file.

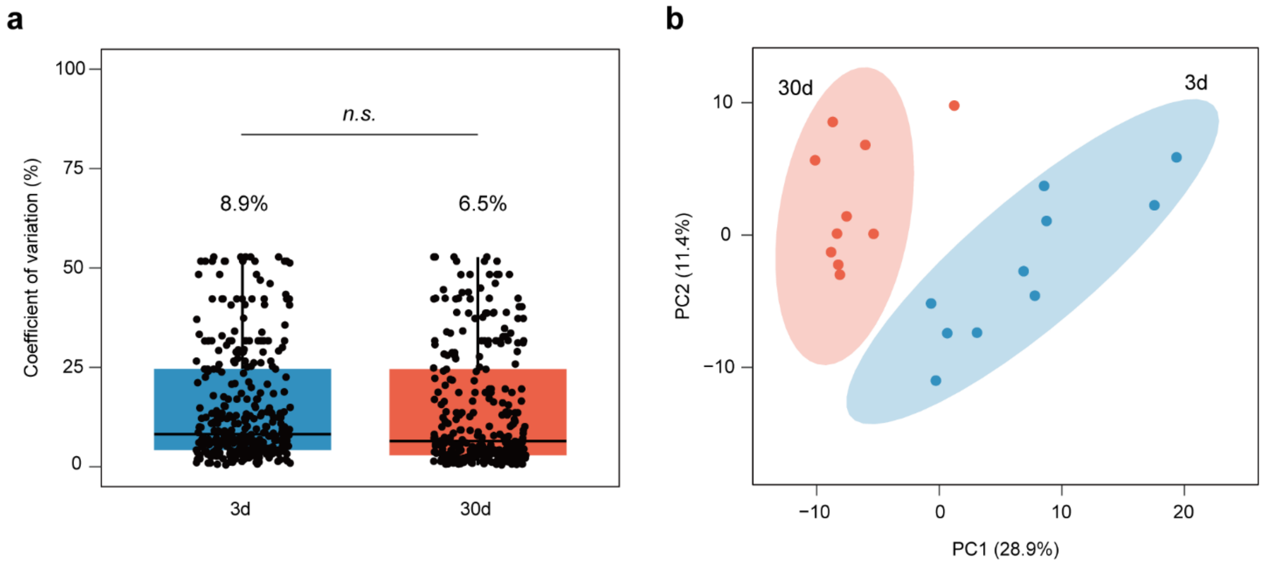

Supplementary Figure 16. Comparison of metabolic heterogeneity between 3d and 30d *Drosophila*. (a) Coefficient of variation (CV) values of labeled metabolites in 3d and 30d flies (n = 390 labeled metabolites; two-tailed Student's *t*-test). The centerlines of the box plots indicate the median; the lower and upper lines in box plots correspond to 25th and 75th quartiles, and the whiskers extend to the most extreme data points within 1.5 interquartile ranges (IQR). **(b)** Principal component analysis (PCA) plot shows the separation of 3d and 30d flies (n = 10 in each group). Colored shadow, 95% confidence interval. Source data are provided as a Source Data file.

Supplementary Table 1

Number of labeled metabolites using four software tools

Dataset	Polarity	MetTracer	El-MAVEN	X13CMS	geoRge
**293T cell (TripleTOF 6600)**	Positive	361	174	166	53
	Negative	469	268	280	139
**293T cell (Exploris 480)**	Positive	245	155	180	75
	Negative	390	188	318	89

Supplementary Table 2

False positive rate evaluation using 293T cell samples acquired on TripleTOF 6600

	MetTracer		El-MAVEN	
**No.of**	Isotopologues	Metabolites	Isotopologues	Metabolites
**False positive**	385	63	775	126
**Total**	10663	1203	6398	719
**FPR**	3.6%	5.2%	12.1%	17.5%

Supplementary Table 3

False positive rate evaluation using 293T cell samples acquired on Orbitrap Exploris 480

	MetTracer		El-MAVEN	
**No.of**	Isotopologues	Metabolites	Isotopologues	Metabolites
**False positive**	231	52	458	95
**Total**	11844	896	8670	571
**FPR**	2.0%	5.8%	5.3%	16.6%

Supplementary Table 4

False positive rate evaluation of MetTracer using Fly head samples acquired on TripleTOF 6600

	Fly		
No. of	False positive	Total	FPR
Isotopologues	309	6368	4.9%
Metabolites	44	670	6.6%

Supplementary Table 5

Names of KEGG pathways for *Drosophila*

KEGG pathway entry	Pathway name
dme00052	Galactose metabolism
dme00500	Starch and sucrose metabolism
dme00564	Glycerophospholipid metabolism
dme00650	Butanoate metabolism
dme00350	Tyrosine metabolism
dme00270	Cysteine and methionine metabolism
dme00630	Glyoxylate and dicarboxylate metabolism
dme00250	Alanine, aspartate and glutamate metabolism
dme00760	Nicotinate and nicotinamide metabolism
dme00340	Histidine metabolism
dme00330	Arginine and proline metabolism
dme00380	Tryptophan metabolism
dme00240	Pyrimidine metabolism
dme00030	Pentose phosphate pathway
dme00053	Ascorbate and aldarate metabolism
dme00040	Phenylalanine, tyrosine and tryptophan biosynthesis
dme00480	Glutathione metabolism
dme00230	Purine metabolism
dme00061	Fatty acid biosynthesis

Supplementary Table 6

Metabolites correlated with pyruvate in 3d *Drosophila*

Node 1	Node 2	*P* value (3d)	*P* value (30d)
Pyruvate	Glycerone	0.029	0.939
Pyruvate	Serine	0.024	0.948
Pyruvate	Succinate	0.008	0.166
Pyruvate	D-Tagatose	0.002	0.877
Pyruvate	L-Galactono-1,4-lactone	0.007	0.857
Pyruvate	Maltose	0.039	0.813
Pyruvate	Tulipalin B	0.011	0.877
Pyruvate	D-Erythrulose	0.003	0.950
Pyruvate	L-Serine	0.024	0.948
Pyruvate	D-Mannose	0.002	0.877
Pyruvate	D-Glucono-1,4-lactone	0.007	0.857
Pyruvate	D-Tagatose_T384	0.002	0.790
Pyruvate	Lactose	0.039	0.813
Pyruvate	L-Erythrulose	0.003	0.950
Pyruvate	D-Allose	0.002	0.877
Pyruvate	D-Galactono-1,4-lactone	0.007	0.857
Pyruvate	Cellobiose	0.039	0.813
Pyruvate	Glucose	0.002	0.877
Pyruvate	L-Sorbose	0.002	0.877
Pyruvate	2,3,4,5-Tetrahydrodipicolinate	0.037	0.313
Pyruvate	D-Tagatose_T404	0.024	0.760
Pyruvate	D-galacto-Hexodialdose	0.007	0.857
Pyruvate	D-Galactono-1,5-lactone	0.007	0.857

(*P* value of Pearson correlation coefficient was calculated by two-sided Student's *t*-test with FDR adjustment)

Supplementary Table 7

Comparison of metabolic observations between our study and other studies

Fly strain	Major metabolic conclusions	Reference	Related conclusions in MetTracer
Oregon-R wild type	In the hypoxia model of *Drosophila*, old flies showed lower TCA cycle fluxes during recovery from hypoxia.	Coquin et al., 2008, Molecular Systems Biology[1]	No related observations in the context of hypoxia.
Canton-S wild type	The number of detected metabolites increased as a function of age, followed by cessation of the increase in late life.	Avanesov et.al., 2014, eLife[2]	Aging influenced the system-wide metabolic coordination.
15 inbred fly lines from DGRP	Glycolysis related metabolites were declined with aging. Carnitines were declined with aging.<br>Metabolites associated with glycerophospholipids showed either increases or declines with age.	Hoffman et.al., 2014, Aging Cell[3]	Consistent with the literature. Glycolysis was down-regulated with aging.<br>No related observations in glycerophospholipids and carnitines.
w1118	Diet restriction (DR) significantly slowed age-related changes in the metabolome and enhanced metabolite correlations with age in *Drosophila*.<br>Amino acids such as leucine, tryptophan and betaine showed declines upon DR, and increased in aging.<br>Beta-alanine biosynthesis, SAM cycle and methionine degradation were downregulated upon DR, and increased in aging.	Laye et.al., 2015, Aging Cell[4]	Metabolite-metabolite correlations were lost during normal aging.<br>Consistent with the literature. Betaine was increased with aging. SAM and SAH were increased with aging. Metabolite precursors of one-carbon metabolism such as serine and glycine were increased with aging.
w1118, yw, Canton-S	Enhancement of SAM catabolism extends the lifespan in *Drosophila.*	Fumiaki Obata, Masayuki Miura, 2015 Nature Communications[5]	Consistent with the literature. SAM and SAH were increased with aging. Metabolite precursors of one-carbon metabolism such as serine and glycine were increased with aging.
w1118	Metabolites such as glutamine, alanine, glycine, tyrosine, tryptophan were highly correlated with the increase of age.<br>Metabolites including leucine, valine, methionine were correlated with sex in aging process.	Zhou et.al., 2017, Experimental Gerontology[6]	Metabolites such as glutamine, alanine and glycine were altered with aging.

w1118	Glycolysis was downregulated with aging, and the activity of glycolysis was enhanced in long-lived PRC2 mutants.	Ma et.al., 2018, eLife[7]	Consistent with the literature. Glycolysis was declined with aging.
B3, O1, O3	Level of tyrosine was increased with age in long-lived flies. Tyrosine supplementation and downregulation of enzymes in the tyrosine degradation pathway significantly extend *Drosophila* lifespan.	Parkhitko et.al., 2020, eLife[8]	No related observations.
178 inbred fly lines from DGPR	Proteinogenic amino acids and metabolites involved in α-KG and glutamine metabolism were correlated with the magnitude of the lifespan response upon diet restriction.	Jin et.al., 2020, PLOS Genetics[9]	α-KG, glutamine, and proteinogenic amino acids such as proline, alanine were associated with aging.
w^{Dah}	High-sugar feeding shortened fly lifespan through increasing purine catabolism.	Dam et.al., 2020, Cell Metabolism[10]	Purine metabolism was observed to be enhanced in old flies.
w^{Dah}, w^{iso31}, Canton-S	Purine metabolism was increased in aged flies, and purine metabolites were impacted by gut bacterial species distinctively.	Yamauchi et.al., 2020, iScience[11]	Consistent with the literature. Purine metabolism was observed to be enhanced in old flies.
Oregon-R	TCA cycle components were decreased with aging.<br>Triacyclglycerols and phospholipids were increased with aging.<br>Amino acids such as histidine, tyrosine and leucine have trends towards lower levels with aging.	Tapia et.al., 2021, International Journal of Molecular Sciences[12]	TCA cycle components such as α-KG and succinate were decreased with aging.<br>No related observation for triacyclglycerols, phospholipids, histidine, tyrosine and leucine
w1118	Decreases in riboflavin and metabolites in purine metabolism were observed in the eyes of aged flies, which were distinct from those observed in the head or whole fly. Metabolites involved in SAM regeneration was increased in the eyes of aged flies.	Hall et.al., 2021, Molecular Cell Proteomics[13]	Purine metabolism was observed to be upregulated during aging in fly head.<br>SAM and SAH were increased with aging.
20 fly lines from DGPR	Metabolites such as proline, glutamate, glutamine, and tryptophan were associated with aging.	Zhao et.al., 2022, Aging Cell[14]	Metabolites such as proline, glutamine and glutamate were dysregulated during aging.

Supplementary Table 8

The parameters for MetTracer

Routine	Parameter
ExperimentParam	ppm = 25
	res.define = 400
	element.trace = 12C
	element.label = 13C
IsotopologueParam	method = “max”
	rt.extend = 15
PeakdetectionParam	peakwidth = c(5,30)
	snthr = 3
	method.peakdetection = “centWave”
	method.smooth = “Gaussian”
ExtractParam	d.extract = “labelled”
	method.align = “apex”
	method.best = “maxint”
	method.quant = “rawinroi”
	nscan.quant = 3
	minfrac = 0.5
	correct.iso = TRUE
	adj.contaminate = TRUE
	thr.contaminate = 0.02

Supplementary Table 9

The parameters for X^{13}CMS, geoRge and El-MAVEN

Tool	Routine	Parameter
X^{13}CMS	getIsoLabelReport()	IsotopeMassDiff = 1.00335<br>RTwindow = 3<br>ppm = 25<br>noiseCutoff = 1000<br>intChoice = maxo<br>alpha = 0.05<br>enrichTol = 0.0
geoRge	PuInc_seeker	fc.threoshold = 1.2<br>p.value.threshold = 0.05<br>PuInc.int.lim = 300
	basepeak_finder	UL.atomM = 12<br>L.atomM = 13.00335<br>ppm.s = 25<br>Basepeak.minInt = 2000
El-MAVEN	Instrumentation	Polarity = Auto Detect<br>Ionization Type = ESI
	Peak Detection	EIC Smoothing Algorithm = Savitzky Golay<br>EIC Smoothing Window = 3 scans<br>Max Retention Time difference between Peaks = 0.2 min<br>Drop top 20% intensities from chromatogram<br>Baseline Smoothing = 20%
	Peak Filtering	Min. Signal Baseline Difference = 3<br>Min. Peak Quality = 0.0<br>Isotope Peak Filtering is same as Peak Filtering: check
	Isotope settings	Report Isotopes: check<br>Isotopic tracer: C13 check<br>Minimum isotope-parent correlation = 0.5<br>Isotope is within [X] scans of parent = 15<br>Link isotope peak RT range with parent peak: Check

El-MAVEN	Alignment	Maximum number of iterations = 10<br>Polynomial Degree = 5<br>At least [X] good peaks in group = 50% samples<br>Limit total number of groups = 1000<br>Peak grouping window = 50<br>Minimum peak intensity = 300<br>Minimum peak S/N Ratio = 3<br>Minimum peak width = 5 scans
	Feature detection selection	Compound database search : check<br>EIC Extraction window = 25<br>Match Retention Time : check 0.25 min<br>Limit number of reported groups per compound = 3 best
	Group filtering	Minimum peak intensity = 300 (Area top)<br>At least = 1<br>Minimum Quality = 0.5 At least = 1<br>Minimum S/B Ratio = 3 At least = 1<br>Minimum S/N Ratio = 3 At least = 50%<br>Minimum peak width = 3 scans<br>Min good peak / Group = 1

Supplementary Table 10

The modifications of default parameters in different software tools.

Software	Parameter	Default	Modified	Reason
$X^{13}CMS$	RTwindow	NA	3 s	Keep the same as MetTracer.
	ppm	NA	25 ppm (QTOF)<br>10 ppm (QE)	Keep the same as MetTracer.
	intChoice	intb	maxo	Keep the same as MetTracer.
	enrichTol	0.1	0	To reduce false positives as recommended in their protocol
geoRge	ppm.s	6.5 ppm	25 ppm (QTOF)<br>10 ppm (QE)	Instrument platform dependent parameter. Keep the same as MetTracer.
ElMAVEN	Min signal baseline difference	0	3	Keep the same as MetTracer
	Minimum isotope-parent correlation	0.2	0.5	At least 50% frequency for the co-occurrence
	EIC extraction window	5 ppm	25 ppm (QTOF)<br>10 ppm (QE)	Instrument platform dependent parameter. Keep the same as MetTracer
	Match retention time	1.0 min	0.25 min	Keep the same as MetTracer.
	Minimum S/N ratio	1 sample	50% of samples	Keep the same as MetTracer.

Supplementary References

1 Coquin, L., Feala, J. D., McCulloch, A. D. & Paternostro, G. Metabolomic and flux-balance analysis of age-related decline of hypoxia tolerance in Drosophila muscle tissue. *Mol Syst Biol* **4**, 233 (2008).

2 Avanesov, A. S. *et al.* Age- and diet-associated metabolome remodeling characterizes the aging process driven by damage accumulation. *Elife* **3**, e02077 (2014).

3 Hoffman, J. M. *et al.* Effects of age, sex, and genotype on high‐sensitivity metabolomic profiles in the fruit fly, D rosophila melanogaster. *Aging cell* **13**, 596-604 (2014).

4 Laye, M. J., Tran, V., Jones, D. P., Kapahi, P. & Promislow, D. E. The effects of age and dietary restriction on the tissue‐specific metabolome of D rosophila. *Aging cell* **14**, 797-808 (2015).

5 Obata, F. & Miura, M. Enhancing S-adenosyl-methionine catabolism extends Drosophila lifespan. *Nat Commun* **6**, 8332 (2015).

6 Zhou, Y. Z. *et al.* Metabonomics approach to assessing the metabolism variation and gender gap of Drosophila melanogaster in aging process. *Exp Gerontol* **98**, 110-119 (2017).

7 Ma, Z. *et al.* Epigenetic drift of H3K27me3 in aging links glycolysis to healthy longevity in Drosophila. *Elife* **7** (2018).

8 Parkhitko, A. A. *et al.* Downregulation of the tyrosine degradation pathway extends Drosophila lifespan. *Elife* **9** (2020).

9 Jin, K. *et al.* Genetic and metabolomic architecture of variation in diet restriction-mediated lifespan extension in Drosophila. *PLoS Genet* **16**, e1008835 (2020).

10 van Dam, E. *et al.* Sugar-Induced Obesity and Insulin Resistance Are Uncoupled from Shortened Survival in Drosophila. *Cell Metab* **31**, 710-725 e717 (2020).

11 Yamauchi, T. *et al.* Gut Bacterial Species Distinctively Impact Host Purine Metabolites during Aging in Drosophila. *iScience*, 101477 (2020).

12 Tapia, A. *et al.* Mild Muscle Mitochondrial Fusion Distress Extends Drosophila Lifespan through an Early and Systemic Metabolome Reorganization. *Int J Mol Sci* **22** (2021).

13 Hall, H. *et al.* Quantitative Proteomic and Metabolomic Profiling Reveals Altered Mitochondrial Metabolism and Folate Biosynthesis Pathways in the Aging Drosophila Eye. *Mol Cell Proteomics* **20**, 100127 (2021).

14 Zhao, X. *et al.* The metabolome as a biomarker of aging in Drosophila melanogaster. *Aging Cell* **21**, e13548 (2022).
