# Supplementary material for: Global stable-isotope tracing metabolomics reveals system-wide metabolic alternations in aging Drosophila
Source: Nat Commun. 2022 Jun 20;13:3518. doi: 10.1038/s41467-022-31268-6 (PMC9209425; doi:10.1038/s41467-022-31268-6)
Supplement: Supplementary file 3 — Description of Additional Supplementary Files [file 41467_2022_31268_MOESM3_ESM.docx]

File Name: Supplementary Data 1

Description: Metabolite annotations of 293T cell dataset acquired on Orbitrap Exploris 480 (input for MetTracer).

File Name: Supplementary Data 2

Description: Metabolite annotations of 293T cell dataset acquired on TripleTOF 6600 (input for MetTracer).

File Name: Supplementary Data 3

Description: Metabolite annotations of *Drosophila* head and muscle tissues (input for MetTracer).

File Name: Supplementary Data 4

Description: Labeled metabolites and isotopologues from four software tools (from 293T cell dataset acquired on TripleTOF 6600).

File Name: Supplementary Data 5

Description: Labeling extent values obtained from MetTracer and manual analysis using Skyline (293T cell dataset).

File Name: Supplementary Data 6

Description: Labeled metabolites and isotopologues from four software tools (from 293T cell dataset acquired on Orbitrap Exploris 480).

File Name: Supplementary Data 7

Descritption: MID and labeling extent (LE) result table of head and muscle tissues for *Drosophila* (3d and 30d).

File Name: Supplementary Data 8

Description: Labeling rate (k value, h-1) result table of head and muscle tissues for *Drosophila* (3d and 30d).

File Name: Supplementary Data 9

Description: RNA-seq result for fly head tissue (3d vs 30d).
