# Supplementary material for: Global stable-isotope tracing metabolomics reveals system-wide metabolic alternations in aging Drosophila
Source: Nat Commun. 2022 Jun 20;13:3518. doi: 10.1038/s41467-022-31268-6 (PMC9209425; doi:10.1038/s41467-022-31268-6)
Supplement: Supplementary file 13 — Reporting Summary [file 41467_2022_31268_MOESM13_ESM.pdf]

nature portfolio

Corresponding author(s): Dr. Zheng-Jiang Zhu

Last updated by author(s): May 23, 2022

Reporting Summary

Nature Portfolio wishes to improve the reproducibility of the work that we publish. This form provides structure for consistency and transparency in reporting. For further information on Nature Portfolio policies, see our Editorial Policies and the Editorial Policy Checklist.

Statistics

For all statistical analyses, confirm that the following items are present in the figure legend, table legend, main text, or Methods section.

n/a	Confirmed	
☐	☒	The exact sample size (*n*) for each experimental group/condition, given as a discrete number and unit of measurement
☐	☒	A statement on whether measurements were taken from distinct samples or whether the same sample was measured repeatedly
☐	☒	The statistical test(s) used AND whether they are one- or two-sided<br>*Only common tests should be described solely by name; describe more complex techniques in the Methods section.*
☒	☐	A description of all covariates tested
☐	☒	A description of any assumptions or corrections, such as tests of normality and adjustment for multiple comparisons
☐	☒	A full description of the statistical parameters including central tendency (e.g. means) or other basic estimates (e.g. regression coefficient) AND variation (e.g. standard deviation) or associated estimates of uncertainty (e.g. confidence intervals)
☐	☒	For null hypothesis testing, the test statistic (e.g. *F*, *t*, *r*) with confidence intervals, effect sizes, degrees of freedom and *P* value noted<br>*Give P values as exact values whenever suitable.*
☒	☐	For Bayesian analysis, information on the choice of priors and Markov chain Monte Carlo settings
☒	☐	For hierarchical and complex designs, identification of the appropriate level for tests and full reporting of outcomes
☐	☒	Estimates of effect sizes (e.g. Cohen's *d*, Pearson's *r*), indicating how they were calculated

Our web collection on statistics for biologists contains articles on many of the points above.

Software and code

Policy information about availability of computer code

Data collection	These tool were used in data collection: Analyst TF Software (version 1.7.1, Sciex, USA), Xcalibur (Version 4.4.16.14, Thermo Fisher Scientific, USA)
Data analysis	The source code of MetTracer is provided on GitHub [https://github.com/ZhuMetLab/MetTracer] and Zenodo [https://doi.org/10.5281/zenodo.6575308]. A detailed description of open and vendor software in this study has been included in the Methods. R package "X13CMS" (version 1.4); R package "geoRge" (version 1.0); GUI software of El-MAVEN (version 0.11.0); Skyline software (version 20.2.0.286); "AccuCor" (version 0.2.4); "MSFinder" (version 3.24); "xcms" (version 1.46.0 for Triple TOF dataset and version 3.12.0 for Orbitrap Exploris 480 dataset); MetDNA (version 1.2.2); "enviPat" (version 2.4);circlize (version 0.2.10);DESeq (version 1.8.3).

For manuscripts utilizing custom algorithms or software that are central to the research but not yet described in published literature, software must be made available to editors and reviewers. We strongly encourage code deposition in a community repository (e.g. GitHub). See the Nature Portfolio guidelines for submitting code & software for further information.

Data

Policy information about availability of data

All manuscripts must include a data availability statement. This statement should provide the following information, where applicable:

- Accession codes, unique identifiers, or web links for publicly available datasets
- A description of any restrictions on data availability
- For clinical datasets or third party data, please ensure that the statement adheres to our policy

The raw metabolomics and RNA-seq data files generated in this study have been deposited in the National Omics Data Encyclopedia under accession code OEP002699 [https://www.biosino.org/node/project/detail/OEP002699]. The RNA-seq data files generated in this study have been deposited in the Gene Expression

Omnibus database under accession code GSE204740 [https://www.ncbi.nlm.nih.gov/geo/query/acc.cgi?acc=GSE204740]. Sequencing reads were mapped to the Drosophila Melanogaster reference genome dm6 [https://www.genome.ucsc.edu/cgi-bin/hgGateway?db=dm6]. The annotation results for all metabolomics datasets are provided in the Supplementary Data 1-3. Source data are provided with this paper.

Field-specific reporting

Please select the one below that is the best fit for your research. If you are not sure, read the appropriate sections before making your selection.

- [x] Life sciences
- [ ] Behavioural & social sciences
- [ ] Ecological, evolutionary & environmental sciences

For a reference copy of the document with all sections, see nature.com/documents/nr-reporting-summary-flat.pdf

Life sciences study design

All studies must disclose on these points even when the disclosure is negative.

Sample size	The experiment of 293T cell in Figure 1 was performed with three biological independent samples. The experiment in Figure 1 for reproducibility evaluation was performed with 6 technical replicates. The experiments of aging Drosophila were performed with ten biologically independent samples for each group. The experiments of PRC2 mutant Drosophila were performed with eight biologically independent samples for each group. No sample-size calculation was performed here. The sample sizes were chosen because they are the commonly used number for most biological studies in metabolomics society.
Data exclusions	No samples were excluded from analysis in this study.
Replication	The 293T cell samples (n=3 biologically independent samples) were used for data analysis in Figure 1. The pooled 293T cell samples (n=6 replicated samples) were used for reproducibility evaluation in Figure 1. The Drosophila head tissue (n=10 biologically independent samples) and Drosophila muscle tissue (n=10 biologically independent samples) were used for data analysis in Figure 2-5.The PRC2 mutant Drosophila head tissue (n=8 biologically independent samples) were used for data analysis in Figure 5.Each sample was analyzed once by LC-MS.
Randomization	For all datasets, samples were assigned randomly to acquire LC-MS data.
Blinding	The investigators were blinded to group allocation during data collection. At the time of sample acquisition and processing, scientists were completely unaware of the sample group. The data analyses were blinded.

Reporting for specific materials, systems and methods

We require information from authors about some types of materials, experimental systems and methods used in many studies. Here, indicate whether each material, system or method listed is relevant to your study. If you are not sure if a list item applies to your research, read the appropriate section before selecting a response.

Materials & experimental systems

n/a	Involved in the study	
x		Antibodies
	x	Eukaryotic cell lines
x		Palaeontology and archaeology
	x	Animals and other organisms
x		Human research participants
x		Clinical data
x		Dual use research of concern

Methods

n/a	Involved in the study	
x		ChIP-seq
x		Flow cytometry
x		MRI-based neuroimaging

Eukaryotic cell lines

Policy information about cell lines

Cell line source(s)	HEK293T (CRL-2925, ATCC)
Authentication	None of the cell lines used were authenticated.
Mycoplasma contamination	The cell lines were routinely tested for mycoplasma contamination. The cell lines used are free of mycoplasma but not described in text.
Commonly misidentified lines (See ICLAC register)	No Commonly misidentified lines were used.

Animals and other organisms

Policy information about studies involving animals; ARRIVE guidelines recommended for reporting animal research

Laboratory animals	Wild type male fruit flies with age 3d and 30d (FlyBase ID: FBst0005905). PRC2 mutant (Pcl c421/+; Su(z)12 c253/+) male fruit flies with age 8d and 30d.
Wild animals	The study did not involve any wild animals.
Field-collected samples	The study did not involve animals collected from the filed.
Ethics oversight	The study did not need ethic oversight.

Note that full information on the approval of the study protocol must also be provided in the manuscript.
